# Comparison of central venous catheter thoracic drainage and traditional closed thoracic drainage following minimally invasive surgery for esophageal carcinoma: a retrospective analysis

**DOI:** 10.1186/s13019-023-02373-4

**Published:** 2023-10-04

**Authors:** Yang Zhao, Yue Ma, Zhixia Bai, Tao Wang, Dong Song, Tao Li

**Affiliations:** 1 Clinical Medical College, Ningxia Medical University, Yinchuan, Ningxia, 750004 China; 2 Department of Surgical Oncology II, General Hospital of Ningxia Medical University, No.804 Shengli Road, Xingqing District, Yinchuan, Ningxia, Ningxia 750004 China; 3 Department of Anesthesiology, General Hospital of Ningxia Medical University, Yinchuan, Ningxia, 750004 China

**Keywords:** Central venous catheter, Esophageal cancer, Minimally invasive surgery, Thoracic drainage

## Abstract

**Objective:**

To compare the effectiveness and safety of central venous catheter thoracic drainage (CVCTD) with traditional closed thoracic drainage (TCTD) after minimally invasive surgery for esophageal cancer.

**Methods:**

We conducted a retrospective investigation of 103 patients who underwent minimally invasive esophageal cancer surgery at our institution between January 2017 and December 2019. Among them, 44 patients underwent CVCTD, while 59 received TCTD. We compared the following outcomes between the two cohorts: drainage volume, duration of drainage, postoperative complications (including pleural effusion, pulmonary infection, atelectasis, anastomotic leakage, etc.), length of hospital stay, and postoperative pain assessment.

**Results:**

No significant differences were observed between the experimental and control groups regarding postoperative thoracic drainage, the timing of postoperative tube removal, or postoperative complications. However, significant disparities were noted in the duration of postoperative hospitalization, drainage tube healing time, and pain threshold among the esophageal cancer patients in both cohorts (p < 0.05).

**Conclusion:**

CVCTD is a secure and potent alternative to TCTD following minimally invasive surgery for esophageal carcinoma. It potentially contributes to reducing the incidence of postoperative complications while curtailing the duration of hospitalization. Additional research is warranted to substantiate these findings.

## Introduction

Esophageal carcinoma, a dire and frequently fatal condition, calls for prompt and effective remedial measures [1–4]. Minimally Invasive Surgery (MIS) has ascended as a preferred methodology in the therapeutic landscape of esophageal carcinoma, offering a host of benefits such as mitigated blood loss, truncated hospitalization span, and accelerated recuperation periods [5]. A challenge intrinsic to MIS is the postoperative administration of sequels, such as pleural effusion and pneumothorax. Thoracic drainage routinely finds its application in mitigating this sequela, with conventional closed thoracic drainage (TCTD) being the customary approach. Pain engendered by the thoracic drainage tube, frequently eclipsed by postoperative complications, is an issue of substantial importance, primarily attributable to the invasive nature of the conventional closed thoracic drainage tube and the dermal incision necessitated for its anchoring [6]. The cardinal role of thoracic drainage in postoperative pain management for esophageal carcinoma is often undermined, warranting more comprehensive attention.

The medical fraternity specializing in minimally invasive surgery is witnessing a paradigm shift in the quest for safer alternatives to conventional closed thoracic drainage (TCTD) or even the elimination of drainage tubes. Consequently, CVCTD has been posited as a potential replacement for TCTD, and antecedent studies have corroborated the safety and viability of routine thoracic drainage via central venous catheters (CVCs) [7–10]. Notwithstanding, contemporary research scrutinizing the comparative efficacy and safety of CVCTD and TCTD in esophageal carcinoma surgery is deficient.

In light of these considerations, this retrospective study is intended to juxtapose the outcomes of CVCTD and TCTD in patients subjected to MIS for esophageal carcinoma. To authenticate this supposition, we have contrived a single-center, randomized controlled trial to probe the influence of these two distinct drainage methodologies on the prevalence of complications, duration of hospital stays, and postoperative pain assessment succeeding an esophagectomy for esophageal carcinoma.

## Materials and methods

### Study design and patient selection

This retrospective study included patients who underwent minimally invasive surgery for esophageal cancer at our institution between January 2017 and December 2019. The hospital’s Ethics Committee backed our inquiry (Ethics number: 2021-12).

#### Preoperative exclusion criteria

**Fig. 1:**
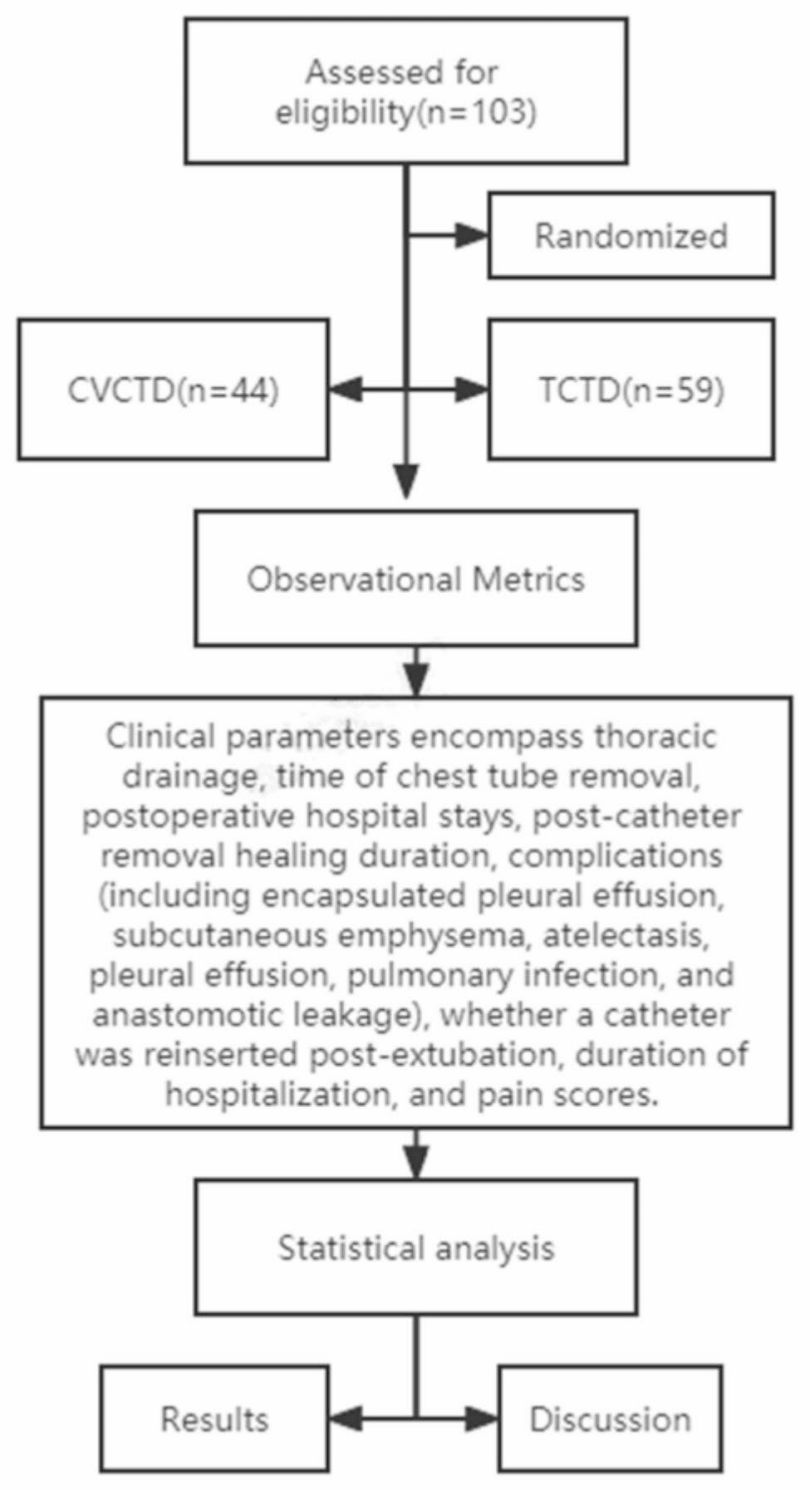
Our flowchart is shown in the figure

 [1] the existence of any unstable systemic underlying conditions, such as active infections, history of tuberculosis, uncontrolled hypertension, or unstable angina; [2] prior history of thoracic surgery; [3] presence of pneumonia or pulmonary atelectasis as revealed by preoperative chest computed tomography (CT) scans; [4] impaired coagulation function; [5] historical usage of anticoagulants; [6] circumstances wherein, due to unpredictable factors (e.g., substantial hemorrhage, severe pleural adhesions), the surgical procedure necessitates a transition from minimally invasive to open surgery. Our process diagram is depicted in Fig. 1.

#### Randomized retrospective study

We endeavored to employ a hybrid methodology of Randomized Retrospective Study, retrospectively reviewing past data or records pertaining to thoracic drainage via central venous catheters or traditional chest tubes following esophageal cancer surgery, and attempting random grouping. Although genuine random assignment was unachievable, we sought to simulate a randomized effect to mitigate potential bias and enhance the reliability of the research outcomes.

### Data collection

Patient demographic data, surgical details, postoperative progression, and complications were collated from electronic medical records. Variables documented encompassed age, gender, body mass index (BMI), tumor location (proximal/mid/distal), pathological type, tumor staging [11], history of smoking, duration of surgery, conversion rate to open surgery, intraoperative blood loss, postoperative drainage, duration of hospital stay, and postoperative complications.

### Thoracic drainage methods

**Fig. 2:**
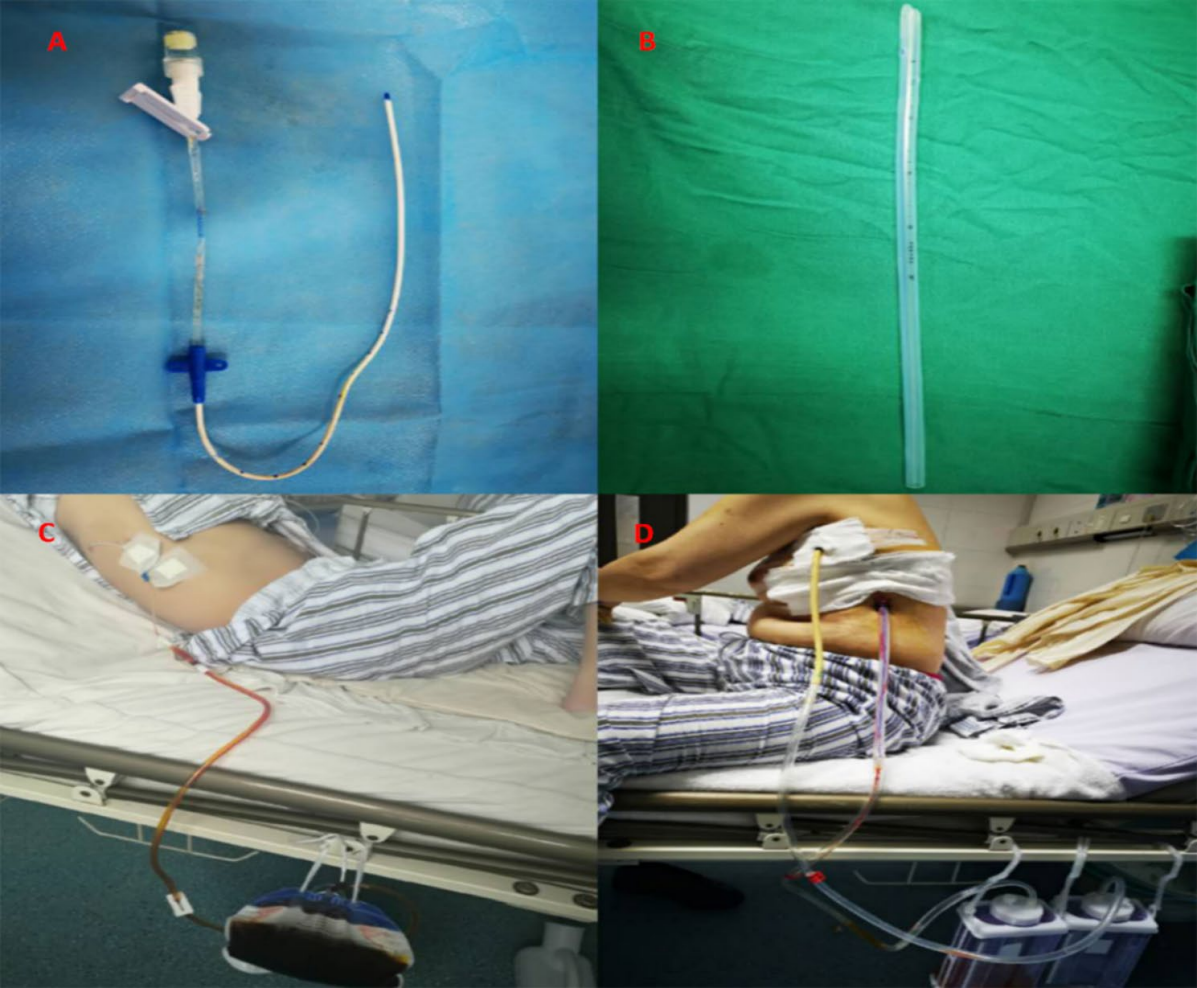
A illustrates the single-lumen central venous catheter (5 F) with a width of 1.6 mm. B shows the silicone tube (16 F) that has a diameter of 5.2 mm. C and D denote the application of the single central venous catheter and the silicone tube drainage respectively, following a surgery for esophageal cancer

This investigation incorporates two variants of thoracic drainage techniques: following the completion of minimally invasive surgery for esophageal cancer, patients are randomly assigned to receive either a central venous catheter thoracic drainage or a conventional closed thoracic drainage. The preference of the attending surgeon dictates the choice of drainage technique. The central venous catheter thoracic drainage is performed by introducing a central venous catheter into the pleural cavity via a small incision under ultrasound guidance. The outer extremity of the Central Venous Catheter (5 F with a width of 1.6 mm, Tuoren, China) is attached to a drainage bag, and to prevent blockage in the lumen, the CVC is regularly cleansed with 20 milliliters of normal saline every eight hours. The conventional closed thoracic drainage (16 F, outer diameter 5.2 mm, Yangzhou, China) is executed by placing a chest tube into the pleural cavity through a small incision. The tube’s outer terminal was linked to a water-sealed drainage container, subject to daily replacement. (Fig. 2).

### Observational metrics

Clinical parameters encompass thoracic drainage, time of chest tube removal, postoperative hospital stays, post-catheter removal Healing duration, complications (including encapsulated pleural effusion, subcutaneous emphysema, atelectasis, pleural effusion, pulmonary infection, and anastomotic leakage), whether a catheter was reinserted post-extubation, duration of hospitalization, and pain scores. Safety was assessed via thoracic CT scans. Upon the appearance of light-yellow thoracic drainage fluid for three consecutive days, a drainage volume of fewer than 150 milliliters, and absence of gas discharge in the thoracic cavity, the central venous catheter or the conventional closed thoracic drainage tube can be removed.

The Numeric Rating Scale is the most widely utilized evaluation tool globally for quantifying postoperative pain. The numeric grading method uses a scale of 0–10 to represent varying degrees of pain. Postoperative pain is evaluated using the Numeric Rating Scale (0–10): 0 signifies no pain; 1–3 denotes mild pain; 4–6 indicates moderate pain; 7–10 signifies severe pain. For mild to severe pain, oral administration of Lofenidine, Paracetamol, Hydrocodone, and Pethidine Hydrochloride is recommended.

### Statistical analysis

Data analysis was performed using SPSS software (version 22.0, IBM Corp., Armonk, NY, USA). Continuous variables were presented as mean ± standard deviation (SD), and categorical variables were presented as frequencies and percentages. Differences in continuous variables between the two groups were analyzed using independent-samples t-tests. Differences in categorical variables were analyzed using chi-squared tests or Fisher’s exact tests. P values < 0.05 were considered statistically significant.

## Results

### Patient demographics and pathologic findings

Blood loss-related data within the baseline statistics of the two cohorts disclosed a statistically significant divergence between the CVCTD and TCTD groups. However, no discernable disparity was observed in other data parameters (Table 1).

**Table 1:** Patient demographics and Pathologic findings

Characteristics		CVCTD (n=44)		TCTD (n=59)	*P value*
*Patient demographics*					
Gender(Male/Female)		24/20	31/28		P=0.840
Age		62.70±8.77	61.98±8.36		P=0.672
Body Mass Index		22.04±3.32	22.46±3.05		P=0.497
Tumor location(Proximal/Mid/Distal)		6/24/14	11/27/21		P=0.644
Smoking history (No/Yes)		20/24	30/29		P=0.588
Operation duration (min)		349.09±44.40	348.81±44.53		P=0.975
Retrieved Lymph nodes		13.11±2.33	13.14±2.67		P=0.965
Blood loss		262.5±87.82	399.8±174.3		P<0.0001
Rates of open conversion		13/44	23/59		P = 0.325
*Pathologic findings*					
Pathologic types (adenocarcinoma/Squamous cell carcinomas)		7/41	9/54		P=0.756
Tumor Node Metastasis	T1/T2/T3/T4	13/8/17/6	13/12/26/8		P=0.849
	N0/N1/N2/N3	18/14/6/6	31/15/7/6		P=0.704

### Contrasting postoperative drainage efficacy in two cohorts of patients afflicted with esophageal Cancer

The findings of our investigation elucidate a notable divergence amongst cohorts concerning the duration of postoperative hospitalization and the continuum of healing subsequent to catheter extraction (P < 0.05). In contrast, no prominent disparities were discerned in other facets (Table 2).

**Table 2:** Comparison of surgical drainage effects in two groups of esophageal cancer patients

Postoperative outcomes	CVCTD(n=44)	TCTD(n=59)	*P value*
Mean drainage volume(ml)	547.72±64.66	536.27±84.67	0.456
Time of chest tube removal (days)	7.61±0.92	7.53±1.02	0.653
Postoperative hospital stays(days)	11.75±2.02	13.03±1.29	**0.000**
Post-catheter removal Healing duration (days)	5.64±1.12	14.02±1.32	**0.000**

### Comparison of postoperative complications between the two cohorts

Our research findings indicate that no significant variation exists between the two cohorts concerning postoperative complications (Table 3).

**Table 3:** Comparison of Postoperative Complications in Two Cohorts

Postoperative Complications	CVCTD(n=44)	TCTD(n=59)	*P value*
Reposition the tube after Extubation	2	7	0.294a
Atelectasis	3	11	0.144 a
Pneumothorax	1	5	0.390 a
Subcutaneous emphysema	2	2	1.000 a
Lung infection	2	7	0.294 a
Anastomotic leakage	3	8	0.345 a

a: Fisher’s Exact Test

### Comparing the NRS pain scores after surgery between two groups of patients diagnosed with esophageal cancer

We compared postoperative Numeric Rating Scale (NRS) pain assessments between the two cohorts. Evidently from Table 3, the Central Venous Catheter cohort reported significantly lower pain levels relative to the conventional cohort (P < 0.05) (Table 3).

**Table 3:** Postoperative NRS pain levels of two groups of esophageal cancer patients were compared

Postoperative NRS Pain score	CVCTD (n=44)	TCTD(n=59)	*P value*
12 h	3.59±0.73	5.14±0.79	0.000
24 h	2.75±0.61	3.51±0.60	0.000
48 h	2.20±0.46	3.22±0.56	0.000
72 h	1.55±0.50	2.59±0.62	0.000
96 h	1.36±0.49	2.46±0.50	0.000

## Discussion

The postoperative employment of closed thoracic drainage tubes in esophageal cancer surgeries has been substantiated to aid in the expulsion of extraneous fluid and gas, invigorate lung lobe recruitment, and mitigate the probability of subsequent pulmonary infections. Despite this, conventional wisdom advocates for the application of broader diameter drainage tubes to augment the proficiency of drainage [12, 13]. Our investigation has uncovered numerous merits of adopting a Central Venous Catheter (CVC) for thoracic drainage in patients subjected to minimally invasive esophageal cancer surgery instead of traditional closed thoracic drainage. These advantages encompass alleviating postoperative pain and discomfort, facilitating patient mobility, thereby rendering substantial benefits to the patient. Although the two groups exhibited no substantial divergence in complications, extubation time, or the volume of thoracic drainage, the duration of postoperative hospitalization and the healing time of the drainage tube was significantly abbreviated in the Central Venous Catheter group, with a remarkably lower intensity of postoperative pain compared to the control group. In this section, we will discuss the findings of our study on the utilization of central venous catheters (CVCs) for thoracic drainage in esophageal cancer surgeries, focusing on their merits, drawbacks, potential complications, and optimal chest tube size [14].

### The merits of central venous catheters in thoracic drainage

Our study builds upon previous research demonstrating the safe and effective use of CVCs in ICU patients, suggesting their potential as an alternative to traditional thoracic drainage methods [15]. We found that CVCs alleviate postoperative pain and discomfort and promote patient mobility [16]. Furthermore, multifaceted research has shown the benefits of CVCs in managing primary or secondary pleural effusion drainage, traumatic hemothorax, and tuberculous pleurisy [7, 17]. A salient study specifically examined using a 7-French CVC as a postoperative pain management alternative to traditional chest tube insertion in patients undergoing thoracoscopic pulmonary lobectomy. The results demonstrated reduced postoperative discomfort, decreased demand for analgesics, and shorter hospitalization duration, providing empirical solid evidence for considering thoracic drainage alternatives following esophageal cancer surgery [18]. Another related investigation involving pulmonary lobectomy identified the prophylactic application of aspiration catheters (CVCs) as a viable and safe alternative to chest tube drainage, with significant reductions in the duration of chest tube usage, length of hospital stays, and incidence of postoperative pneumothorax. These findings align with our study, highlighting the promising potential of CVCs in thoracic drainage [19].

### Potential drawbacks and complications of central venous catheters

While CVCs offer numerous benefits, they are not without potential shortcomings. Various types of these catheters may have small side openings that are susceptible to blockages from fibrous bands in the pleura, compromising their effectiveness [8, 20]. Operative complications during the drainage process, including bleeding, anastomotic leakage, and chylothorax, can also jeopardize patient safety [21–23]. However, the smaller diameter of CVCs, constructed from second-generation polyurethane, ensures superior biocompatibility and resistance to obstruction. In cases of catheter blockage, uncomplicated flushing with normal saline or the use of the accompanying guidewire can restore patency [24]. In contrast, removing an obstructed chest tube presents a challenge, often requiring catheter replacement or repeated cannulation for drainage resumption. Displacement or dislodgement of small-bore chest tubes should also be considered, and the importance of secure fixation through anchoring sutures has been emphasized in previous studies [25]. In our study, we adhered to the standard practice of anchoring sutures during tube placement, successfully preventing tube displacement or dislodgement. Pleural infection is a potential complication following closed thoracic drainage. However, the CVC’s introduction via puncture minimizes disruption to surrounding soft tissues, maintaining a relatively tight seal that prevents blood leakage from the pleural cavity. Soft tissues occlude the puncture hole upon CVC removal, suppressing further blood leakage that could foster bacterial proliferation and migration into the pleural cavity. Prolonged retention of the CVC increases the possibility of drainage fluid leakage, intensifying the risk of infection and potentially leading to empyema during and after the retention period. The risk of infection may be potentially augmented with conventional thoracic drainage tubes [26].

### Optimal chest tube size for drainage

Traditional teaching methods recommend large-diameter chest tubes, but smaller-diameter catheters have recently gained popularity [27, 28]. A comprehensive review of the existing literature supports using smaller caliber catheters. They induce less discomfort while maintaining comparable therapeutic efficacy to their larger counterparts in treating pleural infections, malignant effusions, and pneumothorax [29]. Clinicians should consider using smaller caliber catheters as a less painful and equally effective alternative when treating pleural diseases, aligning with the intent of our investigation. A study conducted a comparative analysis of large-bore and small-bore chest tubes for various conditions such as pneumothorax, primary effusion, and uncomplicated empyema [30]. The results demonstrated that small-bore chest tubes were as effective as large-bore tubes for these conditions. However, small-bore lines may not be as effective for complex empyema and hemothorax [31, 32], which contradicts the findings of a prior study by Yi JH et al. [17]. Therefore, the choice between large-bore and small-bore chest tubes should be based on the specific clinical scenario [33]. In our study, which focused on pleural effusion drainage following esophageal carcinoma surgery, we encountered negligible complications such as hemothorax and complex empyema. Consequently, we opted for small-bore chest tubes (CVCs), resulting in favorable therapeutic outcomes. Furthermore, we conducted an analysis on the data associated with blood loss, revealing statistically significant discrepancies between the CVCTD and TCTD cohorts. This discovery suggests a potential inclination among surgeons to employ conventional thoracic drainage, particularly during surgical procedures that could result in substantial hemorrhage. This notion aligns with the existing literature that indicates a possible occurrence of hemothorax.

### Limitations

As a retrospective study, we employed a hybrid approach of a randomized retrospective investigation, reviewing past data or records and randomly grouping the study subjects. While it was not feasible to genuinely randomize the assignment, we could simulate an impression of randomization to minimize potential bias and augment the reliability of our findings. However, this approach cannot supplant the advantages of randomized trials in terms of causal inference and evidence level; the results may contain inherent biases.

We carried out a renewed analysis of data related to blood loss, revealing a statistically significant disparity between the CVCTD and TCTD cohorts. This discovery implies that surgeons may exhibit a propensity for the utilization of conventional thoracic drainage, particularly during procedures that incur substantial blood loss. Undeniably, inherent limitations are inextricable from a retrospective study. As such, we have endeavored to implement a hybrid approach of randomized retrospective investigation. However, genuinely random allocation remains elusive, which is an inherent shortfall that will be compensated for by prospective randomized controlled trials in future research. Furthermore, the lack of long-term follow-up impedes the evaluation of the sustained benefits of this novel technique for patients. Hence, future studies necessitate larger sample sizes and extended follow-up periods to further substantiate the efficacy and safety of central venous catheter (CVC) technology. Additionally, few clinical studies compare the effectiveness of CVC and traditional chest tubes in esophageal surgeries, which restricts our understanding of their performance in varied circumstances. Therefore, rigorous and extensive clinical trials are required to explore their comparative effectiveness.

## Conclusion

In conclusion, our study highlights the merits of using CVCs for thoracic drainage in esophageal cancer surgeries, including their ability to alleviate pain, promote patient mobility, and offer comparable efficacy to traditional chest tubes. Despite potential drawbacks and complications, such as catheter blockages and operative complications, the use of CVCs provides advantages over conventional drainage methods. Selecting the optimal chest tube size should consider the specific clinical scenario, with smaller caliber catheters proving effective in our study. However, further research with larger-scale studies and longer follow-up periods is needed to validate the findings and establish the safety and effectiveness of CVC technology.
